# Intra-fraction and Inter-fraction analysis of a dedicated immobilization device for intracranial radiation treatment

**DOI:** 10.1186/s13014-020-01639-8

**Published:** 2020-08-18

**Authors:** Chin Loon Ong, Niccolò Giaj-Levra, Luca Nicosia, Vanessa Figlia, Davide Tomasini, Eric M. Franken, Filippo Alongi

**Affiliations:** 1 Department of Radiotherapy, HagaZiekenhuis, Den Haag, The Netherlands; 2 Advanced Radiation Oncology Department, IRCCS Sacro Cuore Don Calabria Hospital, Via Don A.Sempreboni 5, 37024, Negrar di Valpolicella, Verona, Italy; 3 Radiation Oncology Department, ASST Spedali Civili di Brescia, University of Brescia, Brescia, Italy; 4 University of Brescia, Brescia, Italy

**Keywords:** Radiotherapy, Immobilization, Inter-fraction, Intra-fraction, Brain

## Abstract

**Background:**

Immobilization devices are crucial to minimize patient positioning uncertainties in radiotherapy (RT) treatments. Accurate inter and intra-fraction motions is particularly important for intracranial and stereotactic radiation treatment which require high precision in dose delivery. Recently, a new immobilization device has been developed specifically for the radiation treatment of intracranial malignancies. To date, no data are available on the use of this device in daily clinical practice. The aim of this study is to investigate the intra and inter-fraction variations, patient comfort and radiographer confidence of the immobilization system from two distinct institutions: HagaZiekenhuis, Den Haag, Netherlands and IRCCS Ospedale Sacro Cuore Don Calabria, Negrar, Italy.

**Material and method:**

Sixteen patients (10 diagnosed with brain metastases and 6 with primary central nervous systemic tumor) from IRCCS Ospedale Sacro Cuore Don Calabria and 17 patients (all diagnosed with brain metastases tumor) from HagaZiekenhuis were included in this study. The median target volume was 436 cc (range 3.2–1628 cc) and 4.58 cc (range 0.4–27.19 cc) for IRCCS and Haga, respectively. For patients treated in IRCCS Sacro Cuore Don Calabria, the median dose prescription was 30 Gy (range 27–60 Gy) and median number of fractions 10 (range 3–30). In Haga the median dose prescription was 21 Gy (range 8–21 Gy) and the median number of fraction was 1 (range 1–3). The immobilization device was assembled during CT simulation. A short interview to the patient regarding the device’s comfort level was conducted at the end of the simulation procedure. Additionally, simulation setup time and radiographer (RTT) procedures (i.e. mask preparation) were evaluated. Prior to radiation treatment delivery, an automatic rigid match on the cranial bones between cone beam computed tomography (CBCT) and planning-CT was performed. A couch shift was performed subsequently. An extra post-treatment CBCT was acquire after the treatment delivery. This post-treatment CBCT was matched with pre-treatment CBCT to identify any possible intra-fraction motion. All online matches were validated by experienced radiation oncologist or RTT. A total of 126 CBCT’s were analyzed offline by radiation oncologist/medical physicist. The data of the pre-treatment CBCT match was used to quantify inter-fraction motion. The post-treatment CBCT was matched with pre-treatment CBCT to identify any possible intra-fraction motion.

**Results:**

During the molding of the mask, all patients responded positive to the comfort. Median time required by the RTTs to assemble the immobilization system was 9 min (range 6–12 min). In terms of comfort, all patients reported a good-to high level of satisfaction. The RTTs also respond positively towards the use of the locking mechanism and clips. Results of positioning uncertainties were comparable between the two institutes. The mean inter-fraction motion for all translational and rotational directions were < 2 mm (SD < 4 mm) and < 0.5°(SD < 1.5°), respectively, while the mean intra-fraction motions were < 0.4 mm (SD < 0.6 mm) and 0.3° (SD < 0.5°).

**Conclusions:**

This study demonstrates the efficacy and feasibility of the immobilization device in the intracranial radiation treatment. Both patient comfort and preparation time by RTTs are considered adequate. In combination with online daily imaging procedure, this device can achieve submillimeter accuracy required for intracranial and stereotactic treatments.

## Introduction

Accuracy in radiation treatment is considered one of the most relevant issues in modern radiotherapy (RT) [1]. This concept included two distinct aspects, the delivery of high radiation doses to the tumor (e.g. stereotactic cranial and extracranial RT and hypofractionation) and the decrease in normal tissue irradiation. To achieve this balance, several aspects have to be considered: i) the precise definition and verification of the oncological target, supported by radiological and metabolic images (Image guided radiotherapy therapy – IGRT), ii) the use of modern radiotherapy delivery techniques (Intensity Modulated Radiotherapy – IMRT and volumetric modulated arc therapy – VMAT) and finally, during radiation delivery, iii) limiting the inter- and intra-fraction motion with suitable immobilization devices (and, if available, real-time monitoring system such surface guided systems).

All immobilization systems designed for radiation treatment should meet several conditions. The capability of reducing positioning errors and the limiting patient movements alone are not considered sufficient. Good comfort for the patient and short time for the construction of the device by radiographers (RTTs) are also important. One of the most relevant aspects recently explored in the literature was the role of immobilization devices, focusing on intracranial treatments and in particular for stereotactic radiotherapy [2–4]. Most commercially available immobilization devices have been evaluated [5]. Recently, a new immobilization device dedicated to the treatment of intracranial disease, including in particular high precision in stereotactic radiotherapy treatment and patient comfort in conventional intracranial treatments. To date, there are not data about its clinical application and the aim of this study is to investigate the setup accuracy of this device. Furthermore, we also collected user experience for this device based on both patients’ and RTTs’ perspectives.

## Material and methods

In this observational study, we investigated the inter- and intra-fraction variations of the Solstice™ SRS Immobilization System, CIVCO Radiotherapy (Kalona, USA) device for precise intracranial radiation treatments. Moreover, we evaluated patient comfort and the time required for preparation by RTTs for molding the system. Data collected from 2 different institutes, HagaZiekenhuis (Haga), Den Haag, Netherlands and IRCCS Ospedale Sacro Cuore Don Calabria, Negrar, Italy, were analyzed. From both institutions, the inclusion criteria were: a) age > 18 years, (b) diagnosis of oncological brain disease eligible to RT, (c) informed consent. Exclusion criteria were: (a) patients not eligible to RT, (b) claustrophobic patients. Focusing on dose prescription, IRCCS Ospedale Sacro Cuore Don Calabria included patients eligible to standard fractionation or moderate hypofractionation, while HagaZiekenhuis enrolled only patients receiving hypofractionated treatments.

### Immobilization device and CT simulation

The Solstice system comprised of a carbon fiber head support, customizable accuform cushion and thermoplastic mask (**Fig. 1**). The head support allows manual pitch setup errors correction by rotating the screw located at the back of the system. Two RTTs were responsible for the construction of the thermoplastic mask and customizable cushion for each patient. The total set up time was calculated, including all the procedures required: from the recline patient position on the CT simulation couch to preparation of the cushion, molding and cooling down of the mask, and finally the acquisition of CT images. Three distinct landmarks were positioned to the mask (1 frontal and 2 laterals). CT simulation was performed without contrast media and the scan length included the whole brain (with a special resolution of 0.30 mm from both institutions). Slice thickness varied between 1 to 3 mm, depending on different internal treatment protocols.

At the end of each procedure RTTs reported in a specific form, any limitation or problem recorded during the procedure. Specifically, the following procedures have been evaluated: 1) pitch locking level (ease of use, ease of locking indentation and stability of lock), 2) mask clips (ease of use). RTTs had the possibility to mark four different options: poor, fair, good, excellent. After CT simulation, a radiation oncologist interviewed the patient in order to collect information about comfort: 1) Did you have a good comfortable head position to the accuform cushion? 2) Did you have a good comfortable with the mask? 3) Did you feel an extreme mask pressure on your face? 4) Do you feel that the simulation procedure was too long? 5) Extra feedback(s) from the patients. The patients could answered each question by rating it as poor, fair, good or excellent. This information was recorded in the form, with the aim to compare patient comfort and any possible limitation detected by RTTs during the mask preparation. Finally, radiation oncologist conversed with RTTs, in order to record any potential problems observed during the mask procedure. A dedicated homemade questionnaire was created and used from both centers.

### Target volume definition

In IRCCS Ospedale Sacro Cuore, the target volume definition was different according to histology and radiation dose prescription. In high grade gliomas, the gross tumor volume (GTV) was defined as the surgical cavity or residual disease or macroscopic disease detected on T1 sequence on magnetic resonance images (MRI) with contrast. The clinical target volume (CTV) was obtained by adding a 15 mm isotropic margin from the GTV. The planning target volume (PTV) was achieved adding a 5 mm margin to the CTV. For whole brain radiotherapy in multiple brain metastases, CTV was the entire brain and a 5 mm isotropic margin was used to create the PTV. For stereotactic radiation treatment, GTV was defined as the macroscopic disease detected on T1 contrast sequence on MRI. The PTV margin use for IRCCS for stereotactic treatment was 2 mm. In Haga, the GTV was similarly defined using a fusion between T1 contrast sequence on MRI and CT simulation. No CTV margin was used for hypofractionated treatment and an isotropic margin of 1 mm was used to create the PTV. For both institutions, the contouring software provided a dedicated rigid fusion tool between MR images and CT simulation. The rigid fusion was allowed according the cranial immobility. Radiation oncologists had a high level of confidence and experience to evaluate the fusion quality between MR and CT images. In stereotactic radiation treatments, PTV margins used by IRCCS and Haga were justified by DEGRO guidelines [3].

In IRCCS, the dose prescriptions as followed: high grade glioma 60 Gy in 30 fractions (2 Gy per fraction), multiple brain metastases 30 Gy in 10 fractions (3 Gy per fraction), while in stereotactic intracranial treatment, dose prescription was 27 Gy in 3 fractions (9 Gy per fraction). In Haga, all stereotactic patients were treated with 16–21 Gy in 1 fraction or 18 Gy – 25.5 Gy in 3 fractions (6–8.5 Gy), depends on the PTV volume, its proximity to the organs at risk and histology [6].

### Positioning workflow

In IRCCS Ospedale Sacro Cuore Don Calabria, all patients were treated with a TrueBeam™ (Varian Medical System) v. 2.0 Perfect Pitch configuration, due to the high precision in the definition of movement variation in all directions (6D positioning system). In Haga, all patients were treated with using Elekta Synergy Agility linear accelerator in combination with the pitch-rotational functionality available with the Solstice system. The online imaging procedures were slightly different between the 2 institutes.

In IRCCS, a single CBCT was acquired before each radiation treatment. A rigid match between simulation CT and CBCT images was performed automatically by the software, using cranial bones as focus point and validated by a radiation oncologist and RTTs. The setup error tolerance was difference in conventional or SRS treatments. In the conventional fractionation shift tolerance in translational and rotational inter-fraction motions were ≤ 7 mm and 3°, while ≤2 mm and 2° was applied in SRS treatments. In all cases a 6D correction was executed. If the tolerance was exceeded, the patient was repositioned and the entire procedure was repeated. At the end of the session a post-treatment CBCT was performed with the aim to identify patient movements during the treatment delivery (intra-fraction motions).

In Haga, at least 2 CBCT’s were acquired before the radiation treatment. The first CBCT were acquired and a rigid registration was performed based on cranial bone. Subsequently, setup errors in all translational directions were corrected. If a pitch rotational error of > 1° was detected, this would be manually corrected with the Solstice system. The roll and yaw rotational errors were automatically translated into translational corrections in XVI software during the match. The accuracy of this adjustment was verified with a second CBCT. For each radiation delivery, online CBCTs were validated by experienced radiation oncologist or RTT. If all translational and rotational setup errors were smaller than 1 mm/3°, treatment fields will be delivered. The data collection was performed off-line by a medical physicist after the end of each session.

### Patients

Between January 2018 and August 2019, a total number of 126 pre (63) and post treatment (63) CBCTs were analyzed, from 16 and 17 patients with a diagnosis of intracranial oncological disease tumor treated at IRCCS and Haga, respectively. In IRCCS, ten (62.5%) patients had a diagnosis of brain metastases, while 6 (37.5%) reported a primary central nervous systemic tumor. All patient treated in Haga were diagnosed with brain metastases tumor. Median target volume was 436 cc (range 3.2–1628 cc) and 4.58 cc (range 0.4–27.19 cc) for IRCCS and Haga, respectively.

The median dose prescription was 30 Gy (range 27–60 Gy), and 21Gy (range 8 Gy – 21 Gy) for IRCCS and Haga, respectively. The median number of fractions was 10 (range 3–30), and 1 (range 1–3) for IRCCS Calabria and Haga, respectively. The details of patient characteristics and dose prescriptions are listed in Table 1.

**Table 1:** Patient characteristics and dose prescriptions at IRCCS Sacro Cuore Don Calabria and HagaZiekenhuis

	**IRCCS Ospedale Sacro Cuore Don Calabria**	**HagaZiekenhius**
**Number of patients (%)**	16 (100%)	17 (100%)
**Male (%) and female (%)**	7 (43.8%) and 9 (56.2%)	10 (58.8%) and 7 (41.2%)
**Brain metastases cases (%)**	10 (62.5%)	17 (100%)
**Primary CNS tumor cases (%)**	6 (37.5%)	0
**Median target volume (cc) (range)**	436 (3.2–1628 cc)	4.58 (0.40–27.19 cc)
**Median dose prescription (range)**	30 Gy (27–60 Gy)	21 Gy (8–21 Gy)
**Median Number of fraction (range)**	10 (3–30)	1 (1–3)

*CNS* central nervous system

### Set-up error, inter and intra-fraction data collection

The inter-fraction variability was obtained by matching the first CBCT with planning-CT. The same radiation oncologist reviewed off-line the images to confirm the quality of the match, focusing on bone structures and air cavities matching. If the match was suboptimal, a second radiation oncologist will perform a double check.

A standardized off-line procedure has been used to collect data, with the support of ARIA® version 15.1 – Varian™ (IRCCS) and Elekta XVI version 5.0 (Haga). Match values of all three translational axis (x = lateral, y = longitudinal, z = vertical) and three rotational axes (roll, pitch and yaw) from the very first CBCT were recorded in order to establish the daily pre-treatment setup errors (inter-fraction variation). Similar procedure was used to match the post-treatment CBCT to the CBCT acquired right before treatment delivery (intra-fraction variation). Additionally, in order to quantify the deviations in 3D space, a “displacement vector” (D vector) was defined from 3 axes data.

## Results

The feedbacks from all patients and RTTs are presented in Table 2. More than 80% of the patients graded the comfort of the immobilization devices as “Good” and “Excellent”. The median setup time for patients was 9 min (range 6–12 min). And this time is positively experienced by 98% of the patients. RTT did not reported any critical technical issue in the molding and fixation of the mask during all the procedures from simulation to treatment delivery. Most of them reviewed the pitch locking mechanism, stability of the lock and the mask clip very positively. We also did not observe any specific problem with the coach shift after CBCT corrections.

**Table 2:** Patients and Radiographer feedbak on the immobilization device

Feedback	Poor (%)	Fair (%)	Good (%)	Excellent (%)
Patient’s feedback				
Did you feel comfortable with the accuform cushion?	0	18	31	51
Did you feel comfortable with the mask?	0	0	38	62
Did you feel extreme pressure of the mask on your face?	0	2	40	58
Do you feel that the simulation procedure was too long?	0	2	8	90
RTT’s feedback				
Ease of use of the pitch locking mechanism	0	0	30	70
Ease of locking indentification and stability of the lock	0	0	20	80
Ease of use of the mask clips	0	0	0	100

### Inter-fraction variability

Mean and Standard deviation (SD) of the inter-fraction motion for hypofractionated patients from both institutes for all translational and rotational directions are presented in **Fig. 2**. The mean inter-fraction motion for all translational and rotational directions were < 2 mm (SD < 4 mm) and < 0.5°(SD < 1.5°), respectively. The mean 3D-vector displacement of the inter-fraction variability for IRCCS and Haga were 0.23 and 1.18 mm, respectively. The inter-fraction motion for patients treated with normal fractionated schemes were presented in Fig. 4. The mean inter-fraction motion for all translational and rotational directions were < 2 mm (SD < 4 mm) and < 1°(SD < 2.5°), respectively.

### Intra-fraction variability

The intra-fraction mean values were obtained by the match between pre-treatment CBCT and post-treatment CBCT. The mean and SD of the inter-fraction motion for both institutes for all translational and rotational directions are presented in **Fig. 3**. The mean intra-fraction motions for all translational and rotational directions were < 0.4 mm (SD < 0.6 mm) and 0.3° (SD < 0.5°), respectively. The mean 3D-vector displacement of the intra-fraction variability for IRCCS and Haga were 0.13 and 0.26 mm, respectively. The intra-fraction motion for patients treated with normal fractionated schemes were presented in Fig. 4. The mean inter-fraction motion for all translational and rotational directions were < 1.8 mm (SD < 2 mm) and < 0.3°(SD < 0.5°), respectively.

The systematic and random errors for hypofractionated patients were calculated and presented in Table 3. These errors were calculated separately for inter−/ and intra-fraction motions.

**Table 3:** Systematic and random errors for hypofractionated patients

		Systematic errors (mm/°)		Random errors (mm/°)	
		Haga	IRCCS	Haga	IRCCS
Inter-fraction	Longitudinal	0.98	1.59	1.53	1.67
	Lateral	0.61	0.63	1.83	2.10
	Vertical	0.22	0.27	1.23	1.31
	Roll	0.40	0.07	0.77	0.92
	Pitch	0.10	0.19	0.74	1.23
	Yaw	0.24	0.05	1.11	0.96
Intra-fraction	Longitudinal	0.05	0.01	0.23	0.50
	Lateral	0.17	0.10	0.28	0.41
	Vertical	0.19	0.26	0.35	0.17
	Roll	0.01	0.04	0.36	0.19
	Pitch	0.20	0.04	0.24	0.34
	Yaw	0.09	0.06	0.34	0.23

## Discussion

Over the years, several non-invasive stereotactic immobilization system [4–9] and bite blocks [10–12] were introduced. Recently, the Solstice™ immobilization device has been developed and up to date, there are still no data about its clinical application. In this dual centers study, we analyzed the intra and inter-fraction accuracy of the Solstice immobilization system during conventional and stereotactic treatment.

At first, we evaluated patient tolerability and radiographer comfort in the use of this immobilization device. The results of our experience showed that RTTs felt confident with the mask. We also observed a fast learning curve and a progressive decrease in time for mask preparation. In terms of comfort, all patients reported a good-to high level of satisfaction.

The results of inter and intra-fraction variations of both institutes were comparable. For translational and rotational directions, the mean inter-fraction motion was < 1 mm (SD < 4 mm) and < 0.5° (SD < 1.5°), respectively. Daily IGRT procedure, using CBCT, is able to detect patient positioning errors. Hence, these errors are usually corrected before treatment delivery. In terms of treatment accuracy, intra-fraction motions play a more important role. In both institutes, the mean intra-fraction motions for all translational and rotational direction were < 0.2 mm (SD < 0.6 mm) and 0.5° (SD < 0.6°), respectively. This is within the 1 mm PTV margin commonly used for stereotactic radiation treatment [13].

Our results are comparable to current literature on non-invasive stereotactic immobilization systems, despite different measuring and statistical methods were applied [14–21]. One strength point of our approach was the comparison between pre- and post-treatment CBCT. As supported by the literature [14], the use of 6D couch allowed a high precision in detecting positioning variations. In particular, Guckenberger et al. demonstrated that the integration of image guidance significantly affects reducing set-up error from 3.9 ± 1.7 mm to 0.9 ± 0.6 mm [15]. In our experience, the set-up errors were 0.23 mm at IRCCS Ospedale Sacro Cuore Don Calabria and 1.18 mm (3D vector) HagaZiekenhuis respectively, confirming the CBCT accuracy for the isocenter identification.

Analyzing intra-fraction motion, the current literature reported heterogenous results, due to the use of different immobilization system. Intra-fraction 3D vector varied between 0.5 mm to 3.9 mm [15–19]. Our report shows superior intra-fraction 3D vector displacement of 0.13 and 0.26 mm for IRCCS and Haga, respectively. For rotational errors, Babic et al. reported the mean rotational errors between − 0.20° and 0.33° for a variety of immobilization devices [5]. Additionally Nielsen et al. explored the use of 6D Hepadox, intracranial and extracranial radiation treatments. In intracranial treatments, they observed a mean residual rotational setup error of 0.06° (SD 0.3°) [22]. This is similar to our reported mean intra-fraction variabilities of between − 0.2° and 0.47° [5].

The inter-fraction positioning based on stereotactic coordinates is heterogeneous. Accuracy and reproducibility data about patient repositioning varied according to the immobilization system used (with or without bite block). Isocenter deviation varied between 0.5 mm ± 0.7 mm in the experience published by Minniti et al. [6] and 3.7 mm when mask immobilization was used alone [20]. Nevertheless, a more recent article published by Ramakrishna et al. [21] did not record any significant intra-fraction variation in patients treated with radiosurgery using a frame-based versus a frameless image-guided system. Analyzing our data, the use of frameless radiotherapy supported by CBCT was associated with comparable results published in these literatures. We did observed an outlier with a deviation of 10.9 mm in longitudinal direction in one single fraction. This values was observed at the last radiation dose delivery in a patient with a lose weight during the radiation treatment, while the treatment mask consistency was preserved.

The limitations of this study are the limited number of patients enrolled, the heterogeneous population selected, different dose prescriptions and radiation treatment margins. For all patients enrolled in this study, the immobilization device and IGRT procedure remain the same within one institutes. In IRCCS, the inter-fraction uncertainty is larger in patients treated with normal fractionated treatment than hypofractionated treatment. With a longer overall treatment time for normal fractionated treatment, there might be more positioning uncertainties caused by any possible anatomical changes or other time factors. This could lead to larger inter-fraction motion as patients might be positioned slightly differently in the beginning of each treatment. However, once the mask was placed, the patients were firmly immobilized, resulting in comparable intra-fraction uncertainty between the normal fractionated and hypofractionated treatment. And although the IGRT procedures were slightly different between the 2 institutes, the resulting inter−/ and intrafraction uncertainties were comparable as shown in **Fig. 2** and **3**. Due to limited normal fractionated patients enrolled in this study, the systematic and random errors were only calculated for patients treated with hypofractionated schemes. These errors were calculated separately for inter- and intra-fraction motions. A combined systematic and random errors were not calculated as the inter-fraction motions were corrected before the treatment. For intra-fraction motion, both systematic errors and random errors were < 0.3 mm/° and ≤ 0.5 mm/°, respectively.

We demonstrated the potential application of Solstice™ in several clinical scenarios, without any negative impact in intra and inter-fraction values. Additionally, the comparable data resulting from two independent institutions, using two different IGRT procedures, can also further support the reliability and consistency of the performance of the mask, which can be used under different scenarios. Finally, despite limited amount of patients included in this study, the total number of CBCT evaluated is acceptable to support our hypothesis.

## Conclusions

This report showed that Solstice™ SRS Immobilization System, CIVCO Radiotherapy is feasible and efficient for treating patients with intra-fraction lesion. Additional good feedback has been reported by both patients and radiographers. In combination with daily CBCT, the Solstice system could achieve submillimeter positioning accuracy, which is required for high precision stereotactic treatment.

## Data Availability

The patient information may be shared under ‘IRCCS Sacro cuore – Don Calabria’ hospital and HagaZiekenhuis, Den Haag, Netherlands. IRB approval of amendment on a case by case base. The. Solstice™ SRS Immobilization System is proprietary CIVCO Radiotherapy due to patient protection.

## Abbreviations

RT: radiotherapy
SD: standard deviation
IGRT: imaged guided radiotherapy
IMRT: intensity modulated radiotherapy
VMAT: volumetric modulated arc therapy
RTT: radiographers
CBCT: cone beam computed tomography
